# A Hybrid Online Intervention for Reducing Sedentary Behavior in Obese Women

**DOI:** 10.3389/fpubh.2013.00045

**Published:** 2013-10-28

**Authors:** Melanie M. Adams, Paul G. Davis, Diane L. Gill

**Affiliations:** ^1^Department of Physical Education, Keene State College, Keene, NH, USA; ^2^Department of Kinesiology, University of North Carolina at Greensboro, Greensboro, NC, USA

**Keywords:** computer, accelerometer, inactivity, physical activity, waist circumference

## Abstract

Sedentary behavior (SB) has emerged as an independent risk factor for cardiovascular disease and type 2 diabetes. While exercise is known to reduce these risks, reducing SB through increases in non-structured PA and breaks from sitting may appeal to obese women who have lower self-efficacy for PA. This study examined effects of a combined face-to-face and online intervention to reduce SB in overweight and obese women. A two-group quasi-experimental study was used with measures taken pre and post. Female volunteers (*M* age = 58.5, SD = 12.5 years) were enrolled in the intervention (*n* = 40) or waitlisted (*n* = 24). The intervention, based on the Social Cognitive Theory, combined group sessions with email messages over 6 weeks. Individualized feedback to support mastery and peer models of active behaviors were included in the emails. Participants self-monitored PA with a pedometer. Baseline and post measures of PA and SB were assessed by accelerometer and self-report. Standard measures of height, weight, and waist circumference were conducted. Repeated measures ANOVA was used for analyses. Self-reported SB and light PA in the intervention group (I) changed significantly over time [SB, *F*(1, 2) = 3.81, *p* = 0.03, light PA, *F*(1, 2) = 3.39, *p* = 0.04]. Significant Group × Time interactions were found for light PA, *F*(1, 63) = 5.22, *p* = 0.03, moderate PA, *F*(1, 63) = 3.90, *p* = 0.05, and for waist circumference, *F*(1, 63) = 16.0, *p* = 0.001. The intervention group decreased significantly while the comparison group was unchanged. Hybrid computer interventions to reduce SB may provide a non-exercise alternative for increasing daily PA and potentially reduce waist circumference, a risk factor for type 2 diabetes. Consumer-grade accelerometers may aide improvements to PA and SB and should be tested as part of future interventions.

## Introduction

A lack of physical activity (PA) increases the risk of type 2 diabetes among overweight and obese persons and impairs glucose management in those with the disease. Recently, researchers have considered the role of sitting time in cardiometabolic diseases and determined that sedentary behavior (SB) is an independent risk factor (1–4). SB includes time spent sitting at desks, watching television, reading, or commuting (5). Interestingly, breaks from SB have been shown to decrease disease risk (4, 6).

On average, Americans spend 8.44 h a day in SB (4); with obese individuals sitting as much as 2.5 h more than normal-weight individuals (7, 8). A few interventions have been tested to reduce SB and increase light to moderate PA by limiting access to a sedentary activity (9), counting steps (10), or through increased lifestyle PA (11, 12). Lifestyle PA includes tasks of daily living and is less structured than exercise (13), which may be more appealing to overweight or obese women who are not currently physically active.

The hybrid approach combines face-to-face contact with computer-delivered content. This format takes advantage of social influences on behavior and any-time access to the intervention. Computer-delivered interventions appear to be equally effective at increasing PA as traditional methods (14–19). This is a novel approach for reducing SB. Conventional computer use requires participants to sit but also presents an “in-the-act” intervention point. Interest in consumer PA tracking devices such as the Fitbit, Jawbone, or Fuelband, which provide feedback through computer software, makes computer-delivered interventions more relevant.

The aim of this study was to examine the effect of a hybrid intervention for reducing SB on PA, waist circumference, and SB in obese women.

## Materials and Methods

A quasi-experimental, group × time design was used, with participants assigned to either intervention (I) or waitlist-control (WC) conditions. Time spent in SB, light, and moderate PA was measured by self-report (pre-mid-post) and by accelerometer (pre-post). Weekly pedometer steps were tracked in I group. Height, weight, and waist circumference were measured pre and post intervention.

### Participants

Volunteers were recruited from local chapters of a national weight loss support group, Take off pounds sensibly (TOPS™). The chapters were paired and a coin-toss determined I or WC assignments. Four chapters received the intervention (*n* = 40) and three were waitlisted (*n* = 24). No additional chapter was available so the last grouping contained two I chapters and one WC chapter. Women between the ages of 35–85 years, with a BMI > 25 were invited to take part in the study. Participants had to be capable of receiving intervention materials by email and attend all program and data collection sessions. Conditions that prohibited them from standing or walking, such as recovery from surgery, excluded them from the study. TOPS, Inc. is a non-profit organization that offers nutrition, PA, health information, and weight loss tools to members at a low-cost (20). All participants signed the statement of informed consent approved by the university’s Institutional Review Board.

### Measures

#### Objective measurement of SB and PA

Participants wore an Actigraph model GT3X+ tri-axial accelerometer over the right hip (mid-axillary line) during waking hours for 7 days prior to and 7 days immediately following the intervention. The accelerometer recorded the maximum activity count (vector magnitude) in 60 s epochs, providing data on time in light, moderate, and vigorous PA, SB, and steps. Accelerometer data were analyzed using the ActiLife software, version 5.8.3. The cut points were: sedentary (<100 counts), light (101–1951), moderate (1952–5724), or vigorous (>5725) (21, 22). Participants were retained if they had at least 10 h a day of wear time (23) and at least four valid days (24). Sixty minutes of consecutive zero counts was labeled non-wear time (25) and wear periods less than 1 min were ignored (26).

Participants also wore an Advanced Technologies-82 pedometer over the left hip (mid-axillary line) at baseline. Participants used the pedometer for self-evaluation and goal setting during the intervention. Weekly pedometer step counts were collected at four time points during the study (pre, week 3, week 5, post).

#### Self-reported SB and PA

Two recall measures were administered pre, mid, and post intervention. The Godin Leisure-time PA Questionnaire (27) asked participants to recall the number of 15 min bouts of light, moderate, or strenuous PA they engaged in over the last 7 days. The numbers are multiplied by MET values (light 3, moderate 5, strenuous 9), to calculate PA scores. Full scale reliability has been reported as α = 0.74 with lower coefficients for light (0.48) and moderate (0.46) intensities (28). In this sample, test-retest reliabilities were 0.57 for light and 0.44 for moderate. A weekly sitting inventory, taken from Salmon et al. (29), asked for the number of hours and minutes participants engaged in specific SBs (watching TV or video, using computer or internet, reading, socializing, riding in a vehicle, and doing crafts or hobbies) over the past 7 days. This measure has established intra-class reliability (ICC = 0.79. 0.53) (23, 29). The ICC reliability in the current study was 0.62.

#### Anthropometric measures

A Registered Nurse, blinded to group assignment took the height, weight, and waist circumference measures pre and post. Height and weight were converted to Body Mass Index (BMI) using the equation, kg/m^2^. Waist circumference was measured at the narrowest part of the trunk between the iliac crest and last rib (30) with a Gulick measuring tape. Waist circumference was taken twice and the average was recorded.

### Procedure

Due to a limited number of accelerometers, participant chapters entered the study on a staggered schedule. Intervention chapters and WC chapters were paired and observed simultaneously. When possible, chapters were matched according to member and chapter characteristics (email use, meeting schedule, and number of members).

### Intervention

*On Our Feet* was a 6-week intervention framed in the Social Cognitive Theory that targeted self-efficacy for daily PA. Specifically, goal progress was re-enforced with individualized feedback and peers modeled less SB. The intervention was delivered in a combination of face-to-face sessions and email messages. Weeks 1 and 2 were led in-person by the researcher. Weeks 3–6 were conducted by email. Table 1 shows the contacts and measures for each group.

**Table 1:** **Study contacts and measures**.

	Pre	Week 1	Week 2	Week 3	Week 4	Week 5	Week 6	Post
I	Accelerometer	Group session	Group session 1 email	2 Emails	1 Email	1 Email	1 Email	Accelerometer
	Pedometer	Godin SB recall		Godin SB pedometer		Pedometer		Pedometer
	BMI							BMI
WC	Waist circum	Godin SB recall						Waist circum
								Godin SB recall

*I, intervention chapters*.

*WC, waitlisted-control chapters*.

In week 1 the concept of SB as a cardiometabolic risk factor was introduced and as group participants brainstormed alternatives to sitting. Participants received a workbook with weekly logs for steps and sitting time as well as instructions and suggestions to break up sitting time. In week 2 participants received their accelerometer-determined percentages of SB and PA. This feedback along with their week 1 pedometer data was used to develop two goals: (1) to increase breaks from sitting in the next week, and (2) to increase daily steps by week 5. Participants set the goals while guided by the researcher to list specific actions and cues to help reach the goals.

Seven emails contained the computer-delivered content. The messages consisted of either goal reminders, goal feedbacks, or examples of less SBs. All emails were individualized using information from the participant’s goal plan and worksheet. Examples of less SB included short video of a relevant peer modeling the behavior. In week 3 (mid-point), participants completed the Godin Leisure-time Physical Activity Questionnaire and the weekly sitting inventory measures online.

### Data analysis

Group × Time (pre-post) repeated measures analysis of variance (ANOVA) was used to compare I and WC for accelerometer-determined percentage of time spent in SB, light or moderate PA. Self-reported SB and PA data were also analyzed with a Group × Time (pre-post) ANOVA. Only group I completed SB and PA questionnaires at mid-point and a one-way ANOVA was conducted with those data. WC comparisons were made using a repeated measures Group × Time (pre-post) ANOVA. A one-way ANOVA was performed on the I group pedometer step data. Statistical significance was set at ρ ≤ 0.05.

## Results

Sample characteristics are available in Table 2. Participants were mostly White, over age 50, and possessed at least a high school education. Mean BMI at baseline was 36.44 (SD = 7.7). Eighteen participants met the criteria for class I obesity (BMI 30–34.9), 12 for class II (BMI 35–39.9), and 18 were in class III (BMI ≥ 40) (31). Nearly all (96.86%) participants had a waist circumference greater than 88 cm, a level associated with increased risk of cardiometabolic diseases (32). An equal percentage of drop-outs occurred in both groups (14%); drop-outs did not differ significantly in age, health risk, or rural location from those that remained.

**Table 2:** **Sample characteristics**.

	I, *n* = 40	WC, *n* = 24
Age (years)	56.73 (±12.64)	61.38 (±12.1)
BMI (kg/m^2^)	36.37 (±8.19)	36.56(±6.96)
Ethnicity
White	36 (90%)	21 (88%)
African-American	4 (10%)	3 (13%)
Education
<High school	1 (2%)	2 (8%)
High school	15 (38%)	12 (50%)
College or trade school	19 (48)	8 (33%)
Graduate school	5 (13%)	2 (8%)
Employment
Full-time	22 (55%)*	5 (21%)
Part-time	3 (8%)	5 (21%)
Retired	9 (23%)	8 (33%)
Disabled	6 (15%)	6 (25%)
Non-sedentary job	11 (28%)*	5 (21%)
Rural location	18 (45%)	6 (25%)
Membership years	6.31 (±6.91)	4.95 (±5.52)
Cardiovascular disease	16 (40%)	12 (50%)
Type 2 diabetes	16 (40%)	13 (54%)
Arthritis	3 (8%)	4 (17%)
Depression	3 (8%)	4 (17%)
Waist circumference > 88 cm	38 (95%)	24 (100%)

*I, intervention chapters*.

*WC, waitlisted-control chapters*.

*p < 0.05.

### SB and PA

The Group × Time ANOVA showed no significant changes over time or differences between the I and WC groups for the accelerometer-determined SB or PA. The Group × Time ANOVA for self-reported SB and PA, however, did reveal change.

**Figure 1:**
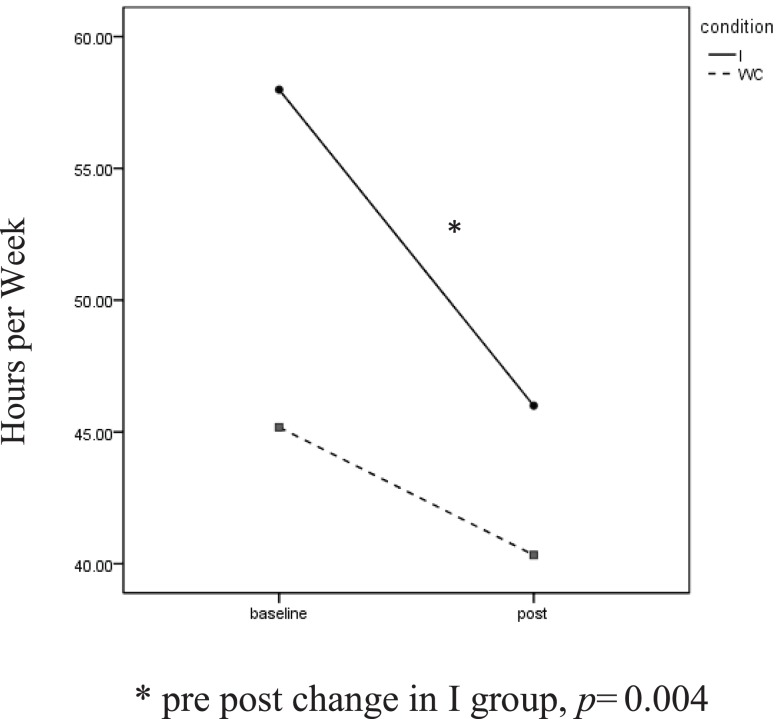
**Self-reported sedentary behavior**. *Pre post change in group, *p* = 0.004.

Self-reported SB showed a significant effect for time, *F*(1, 63) = 4.88, *p* = 0.03, η*p*^2^ = 0.59. Intervention participants reported sitting for 57.9 (SD = 29.7) h a week at baseline. This dropped to 45.9 (SD = 28.91) h at the post assessment. The change was not as great in the WC, decreasing from 45.2 (SD = 34.88) to 40.3 (SD = 4.68) h a week. Paired *t*-tests found the reduction to be significant among I participants, *t*(1, 39) = 3.08, *p* = 0.004, but not for WC participants (Figure 1).

**Figure 2:**
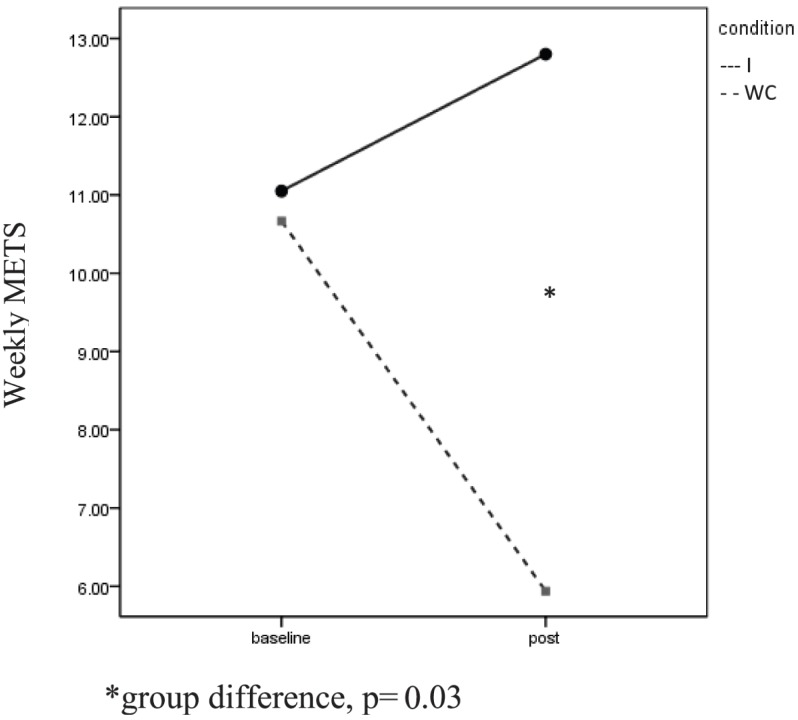
**Self-reported moderate physical activity**. *Group difference, *p* = 0.03.

Significant Group × Time interactions were found for self-reported light PA, *F*(1, 63) = 5.22, *p* = 0.03, η*p*^2^ = 0.61, and self-reported moderate PA, *F*(1, 63) = 3.90, *p* = 0.05, η*p*^2^ = 0.49 (Figure 2). In each case, the I group reported increased PA while the WC participants reported less PA. Independent *t*-tests revealed a significant difference in moderate PA at post between the groups, *t*(1, 62) = 2.27, *p* = 0.03.

**Figure 3:**
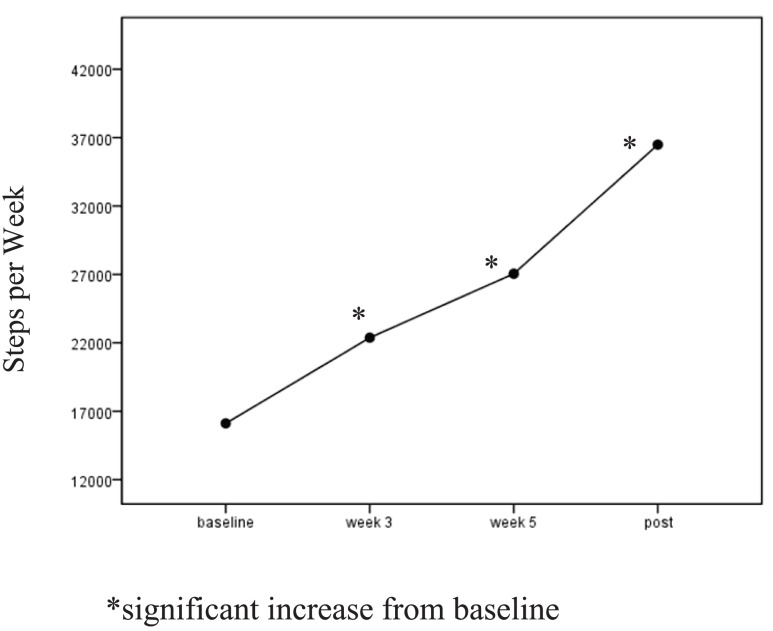
**Intervention pedometer steps**. *Significant increase from baseline.

A one-way ANOVA for the I group revealed significant time (pre-mid-post) effects for SB, *F*(2, 39) = 3.81, *p* = 0.03, η*p*^2^ = 0.09, and for light PA, *F*(1, 2) = 3.39, *p* = 0.04, η*p*^2^ = 0.09. I participants reported decreasing their weekly sitting time from *M* = 57.99 (SD = 29.70) hours to *M* = 49.56 at mid-point and to *M* = 45.99 (SD = 28.91) at post. Self-reported light PA increased from *M* = 9.2 (SD = 11.92) METS per week to *M* = 18.79 (SD = 23.92) by mid-point and regressed to *M* = 12.66 (SD = 15.26) METS at the post assessment. I participants increased their weekly pedometer steps significantly, *F*(1, 3) = 4.3, *p* = 0.006, η*p*^2^ = 0.10. Follow-up *t*-test showed a significant increase in steps from baseline to week 3, *t*(1, 39) = −4.74, *p* = 0.001, and from baseline to week 5 *t*(1, 39) = −4.91, *p* = 0.001. Pedometer steps were not significantly different from week 5 to post (Figure 3).

### Anthropometric measures

**Figure 4:**
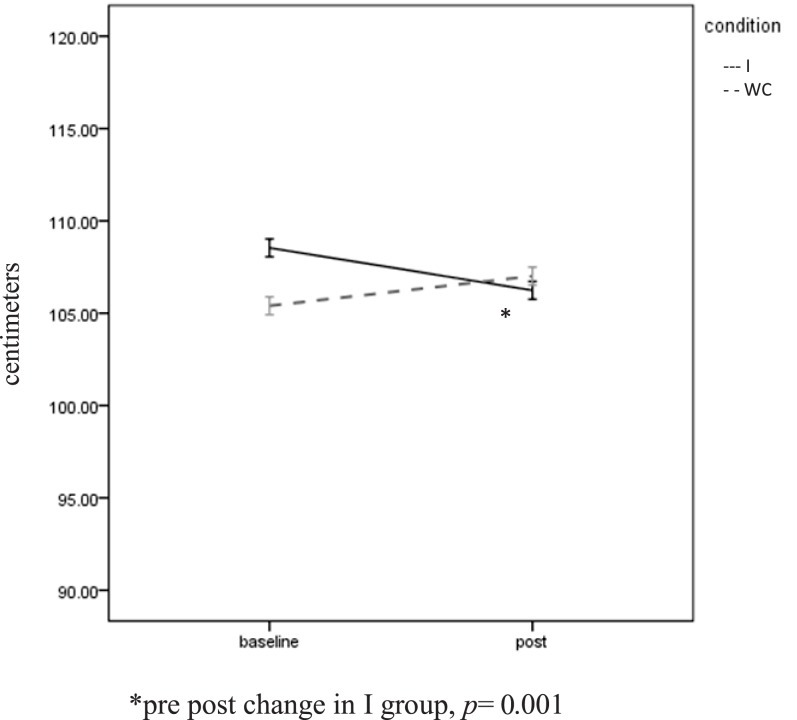
**Waist circumference**. *Pre post change in I group, *p* = 0.001.

A significant Group × Time interaction was found for waist circumference, *F*(1, 63) = 16.0, *p* = 0.001, η*p*^2^ = 0.21. The I group dropped significantly from 108.5 (SD = 15.91) cm to 106.24 (SD = 15.82) cm, *t*(1, 39) = 5.09, *p* = 0.001. A non-significant increase (105.40 ± 13.52 to 107.01 ± 13.07 cm) was seen in the WC group (Figure 4). Twenty-nine of the 40 (72.5%) I participants experienced a reduction in waist circumference. The mean decrease was 2.25 (SD = 2.84) cm. BMI was unchanged over time (36.44 ± 7.70 to 36.48 ± 7.85) and did not differ between the groups.

## Discussion

Self-report data and I pedometer steps point to an increase in PA and reduction in SB over the intervention. Weekly sitting decreased in the I participants at the mid-point with no significant differences between the mid-point and post assessments. Self-reported light PA peaked at mid-point and regressed by the post assessment. While it’s unfortunate that a pre-post change was not seen in the accelerometer counts, it does not mean that the hybrid intervention was not effective. It’s reasonable to conclude that behavior changes were made prior to the post assessment and missed because the accelerometer was only used pre and post intervention.

The significant reduction in waist circumference is further evidence of increased movement in the I group. Since no change in body weight occurred, the decrease in waist circumference was likely due to increased PA rather than calorie restriction. This finding reflects increased energy expenditure over the course of the intervention, whereas the accelerometer data only reflects the last 7 days of the intervention. Body fat redistribution, resulting in reduced waist circumference has been reported without significantly decreased body weight following aerobic exercise training (33).

The improvement in waist circumference is promising. While a small effect, the change came without increases in structured PA, aka exercise. Interventions that encourage more energy expenditure, whether through exercise, household chores, or standing, are a priority for health educators and researchers. The barriers to regular PA are many for obese women, including time, higher rates of perceived exertion, low self-efficacy, and lack of enjoyment (34). Suggesting that inactive persons sit less may overcome these. In follow-up surveys, participants reported high levels of satisfaction with *On Our Feet*, and the combination of face-to-face sessions and email messages was viewed positively.

The ability to self-monitor movement and structure the built-environment is important to changing SB. Participants were frustrated by the inaccuracy of the pedometer; for many the pedometer did not rest vertically on the waistband and steps did not register. *On Our Feet* used pedometers, but a consumer PA tracking device, such as the Fitbit, Jawbone, or Fuelband would have been a better choice for self-monitoring. These PA tracking devices are low-cost accelerometers that detect changes in speed and direction rather than hip vertical displacement as a pedometer does. These devices are more versatile and can be worn at the wrist or clipped to the waist or bra. Particularly for overweight and obese populations, the accelerometer offers more precise measurement of PA (35). An additional benefit of the Fitbit, Jawbone, or Fuelband is the constant feedback that is provided via their software programs. Users are able to sync their device to a computer and track multiple PA variables. They receive messages that positively reinforce improvements, much like the intervention tested here. Unfortunately, these PA tracking devices do not detect standing (versus sitting) and therefore do not help people that wish to monitor their SB.

Also worth noting, both groups engaged in less SB than expected for their age and BMI. Tudor-Locke (36) and colleagues found that obese adult women sat 57.6% of their monitored day. Prior work by Matthews (37) showed that the average daily SB for U.S. Caucasian women aged 40–59 years is 7.74 h (37). At baseline, participants were sedentary for 6.03 (±1.95) h out of 11.65 (±2.16) h or 52% of their monitored time. The fact that 18 I participants improved an average of 6.1% is remarkable given the low prevalence of SB. More research is needed to determine what the rates of SB are for obese persons specific to their occupations and urban or rural environments. Thirty-eight percent of participants lived in rural settings as categorized by the US Department of Agriculture (38) and could explain, in-part, the different levels of SB.

In terms of behavior change, participants found it hard to stand in environments where sitting was the norm. Working at a desk, attending a meeting or being in a waiting room were seen as non-negotiable barriers. More research is needed to determine if offering standing options, especially in the work environment, impact SB. Computerized alarms, that alert workers to the need stand and move are another area to pursue.

### Limitations

Due to accelerometer availability, PA counts were only assessed during the first and last weeks of each intervention period. Had all participants worn the accelerometers over the entire course of the study, a better picture of their SB and PA would have emerged. The self-report measures and pedometer data point to an increase in PA in the I group.

Accelerometer wear time was lower in this study than in the cited research. Participants in the Tudor-Locke (36) and Matthews (37) cohorts wore the accelerometer for an average of 13.8 and 13.9 h a day. Wear time in this study was about 2.25 h short of these standards. While 10 h of daily wear is considered valid (23), lower wear times have been shown to impact SB, both inflating and deflating accelerometer estimates (25). Possibly the lower wear time in this study accounts for the differences in SB noted between this sample and the national data.

Another limitation is that no dietary measures were used to ensure similar pre and post calorie intakes. While no change in weight was observed, as members of a weight loss program, participants could have altered their diet and contributed to the reduction in waist circumference. Alternatively if participants increased their intake, any energy expenditure from increased PA would have been offset so that weight would remain constant. Study participants were long-time members of TOPS (*M* = 5.8 years) and were less likely to make dietary changes than new members.

### Summary

A short trial of a hybrid intervention to reduce SB in obese women was promising. Intervention participants increased self-reported PA and reduced self-reported SB as compared to the waitlisted-control group. They experienced the additional health benefit of reduced waist circumference. New PA tracking devices that combine accelerometers with real-time feedback may be useful in future SB and PA interventions. The role of the built-environment and programmable alerts should also be tested.

## Conflict of Interest Statement

The authors declare that the research was conducted in the absence of any commercial or financial relationships that could be construed as a potential conflict of interest.
